# A Yeast Metabolite Extraction Protocol Optimised for Time-Series Analyses

**DOI:** 10.1371/journal.pone.0044283

**Published:** 2012-08-29

**Authors:** Kalesh Sasidharan, Tomoyoshi Soga, Masaru Tomita, Douglas B. Murray

**Affiliations:** Institute for Advanced Biosciences, Keio University, Nipponkoku 403-1, Daihouji, Tsuruoka City, Yamagata, Japan; University of Kent, United Kingdom

## Abstract

There is an increasing call for the absolute quantification of time-resolved metabolite data. However, a number of technical issues exist, such as metabolites being modified/degraded either chemically or enzymatically during the extraction process. Additionally, capillary electrophoresis mass spectrometry (CE-MS) is incompatible with high salt concentrations often used in extraction protocols. In microbial systems, metabolite yield is influenced by the extraction protocol used and the cell disruption rate. Here we present a method that rapidly quenches metabolism using dry-ice ethanol bath and methanol *N*-ethylmaleimide solution (thus stabilising thiols), disrupts cells efficiently using bead-beating and avoids artefacts created by live-cell pelleting. Rapid sample processing minimised metabolite leaching. Cell weight, number and size distribution was used to calculate metabolites to an attomol/cell level. We apply this method to samples obtained from the respiratory oscillation that occurs when yeast are grown continuously.

## Introduction

A comprehensive *in vivo* understanding of the underlying dynamics of metabolite reaction networks, enzyme kinetics and signalling requires the precise characterisation of intracellular metabolites at specific time points. Recent advances in high-throughput mass-spectrometry allow for the detailed metabolome-wide analysis with high accuracy [1]–[4]. However, development of metabolite extraction protocols has generally lagged behind detection methods. These protocols often suffer from complex experimental design (making time-series analysis difficult), metabolite leakage during processing, metabolite oxidation during sampling/extraction [5], [6], and metabolite specificity (acid-stable or alkali-stable metabolites). Furthermore, yields are influenced by the metabolic state (growth phase and rate) and properties of the species and/or strains used [7]–[10]. This leads to the paradoxical situation where the extraction protocol dictates the experimental conditions. An ideal metabolite extraction protocol should rapidly sample and quench the underlying metabolic processes, i.e., minimise degradation and modification of metabolites, and have a high and reproducible yield [7], [10], [11].

Turnover rates of metabolic intermediates change in the order of seconds and are highly sensitive to the changes in external conditions, thus rapid quenching is required [10], [12], [13]. Therefore, direct centrifugation or filtration of live cells prior to quenching should be avoided as they may alter the metabolite profile [7], [10]. However, the removal and subsequent analysis of the culture media is highly desirable and must be done rapidly to avoid interfering with intracellular metabolite concentrations [5], [7], [10]. Furthermore, the culture media may contain a sufficiently high concentration of salts that may suppress signals or interfere with the chromatography/electrophoresis stage.

There are several methods widely used for extracting metabolites from yeast cells such as freeze-thaw, sonication, hot water, boiling ethanol, permeabilisation using chloroform and treatment with extreme pH. However, these methods are mostly optimised and tested only on fast-growing low-density laboratory strains [9], [11]–[13] and the extraction buffers often use salts that are not compatible with capillary electrophoresis mass spectrometry (CE-MS). Moreover, non-laboratory strains are usually nutrient limited, slow-growing and recalcitrant to lysis. This resistance arises from changes in the cell wall structure [14], [15]. These physiological changes lead to differences in metabolite extraction efficiency and reproducibility, which are critical factors for the analysis of cultures grown in different conditions.

A combined chemical-mechanical disruption by bead-beating using zirconia/silica beads has a high and consistent cell disruption efficiency independent of respiratory state and the cell division cycle [16]. It has also been observed that the metabolic reactions of *Saccharomyces cerevisiae* can be efficiently quenched in methanol (final methanol concentration >50% v/v) at −40°C and having very low metabolite leaching when the media is removed [5], [12]. Moreover, extraction with chloroform-methanol has been used for the quantitative metabolite extraction of rapidly-growing, glucose-repressed laboratory strains of *S. cerevisiae* [9], [11], [12], [17]. The permeabilisation technique used was time consuming (45 min) and it is known that many laboratory strains have been selected for their ability to be easily disrupted. Any metabolite modifications happening during the extraction procedure can be avoided partially by keeping a low temperature (<−20°C) throughout the extraction process [10]. However, oxidation of metabolites remains an issue, for example, precise quantification of the redox sate of thiol groups are critical for understanding redox biochemistry *in vivo* [18]. However, oxidation of thiol groups during extraction usually hinders the accurate determination of redox state [6].

**Figure 1:**
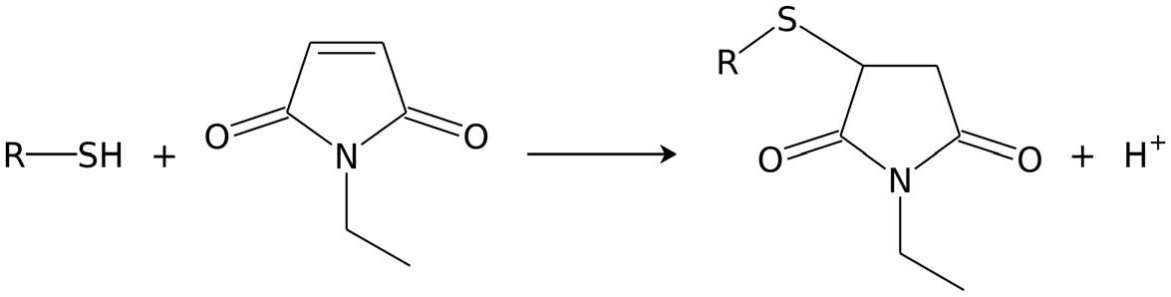
The general reaction scheme for *N*-ethylmaleimide on biological thiols. The product formed is an *N*-ethylsuccinimido conjugate and the monoisotopic mass of the reactant is increased by 125.048. For example the glutathione (m/z 308.0911) conjugate is *N*-ethylsuccinimido-S-glutathione (m/z 433.1391).

Previously we developed efficient methods for the disruption of budding yeasts for mRNA, proteins and DNA [16]. We optimised these methods for metabolite analyses that rapidly extract and fractionate the intracellular and extracellular metabolites. Our method was based on the rapid quenching of cell cultures with −80°C methanol and *N*-ethylmaleimide (NEM) solution. Here, NEM was used to protect thiols from oxidation by binding to -SH groups (figure 1) [19]. The quenching solution (which contains the extracellular metabolites) was rapidly removed and lyophilised. Cell pellets were bead-beated in chloroform/methanol/internal standard (IS) solution. Intracellular and extracellular metabolites were analysed using CE-MS. In parallel, we fixed a sample in ethanol to determine dry cell weight and cell number. We tested our method on continuously grown industrial *S. cerevisiae* cultures and it outperformed other tested extraction techniques (freeze-thaw and sonication), protected the redox state of the cell, has the potential to cover a large fraction of the known yeast metabolome, and gave comparable yields to those reported values for extracted metabolites from cells grown under similar conditions.

## Methods

### Strain and Culture Conditions

Unless stated otherwise, all chemicals were supplied by Wako Chemicals, Japan or Fisher Chemicals, UK. In this study we used IFO 0233 diploid strain of *Saccharomyces cerevisiae*. The colonies were maintained at 4°C on yeast extract peptone dextrose (YEPD) agar plates, comprising 10 g/L yeast extract (Becton Dickinson, Japan/UK), 20 g/L glucose monohydrate, 20 g/L mycological peptone (Becton Dickinson, Japan/UK) and 30 g/L agar (Becton Dickinson, Japan/UK). Pre-cultures (10 mL; 0.5×YEPD broth) were inoculated with yeast colonies and incubated (30°C) in an orbital incubator (250 rpm) for 48 h. The basic medium used was composed of 5 g/L of (NH_4_)_2_SO_4_, 2 g/L of KH_2_PO_4_, 0.5 g/L of MgSO_4_.7H_2_O, 0.1 g/L of CaCl_2_.2H_2_O, 0.02 g/L of FeSO_4_.7H_2_O, 0.01 g/L of ZnSO_4_.7H_2_O, 0.005 g/L of CuSO_4_.5H_2_O, 0.001 g/L of MnCl_2_.4H_2_O, 0.5 mL/L of 70% H_2_SO_4_, 1 g/L of yeast extract (Becton Dickinson, Japan), 0.2 mL/L of Antifoam A (Sigma, USA) and 20 mL/L ethanol [20]. Cells were grown as described previously in a modified version of a MBF-250 bioreactor (Eyela, Japan), at 30°C (±0.02°C), pH 3.4 (±0.03), an aeration rate 0.150 L/min (±0.02 L/min), a working volume of 650 mL, 750 rpm (±3 rpm) agitation rate and the reactor pressure was maintained below 375 Pa, above atmospheric pressure [21]. Prior to initiating continuous culture mode, batch cultures were starved in fermentors for at least 6 h. Stable autonomous respiratory oscillation occurred 16 to 48 h after the initiation of the continuous culture (dilution rate at 0.087 h^−1^) and the respiration was monitored by measuring the dissolved oxygen (DO) content in the culture using an immersed polarographic electrode (InPro6800, Mettler Toledo, Japan/UK). As we could not measure a change in biomass or cell number during continuous growth (biomass was in steady state), this dilution rate equates to a cell doubling time of 8.1 h [22].

### Metabolite Extraction (figure 2)

#### Sampling and quenching

To study the reproducibility (table 1) all samples were taken at the minimum dissolved oxygen concentration during the oscillation. Whereas, time-series samples were obtained at 4 min intervals over two oscillation cycles. To avoid any dead volume in the sampling needle 200 µL was removed 5 s prior to obtaining 0.7 mL of sample (gas and liquid) and the needle end of the syringe sealed. To rapidly degas the sample the plunger was removed rapidly and 500 µL of sample was rapidly pipetted into 1 volume of freshly prepared NEM methanol solution (4 mM) kept in screw cap tubes (1.5 mL; Simport, Canada) equilibrated to −70°C using dry ice/ethanol bath. The samples were then pelleted by flash centrifugation (20,000 g; −9°C) and the supernatant was transferred into fresh 1.5 mL tubes to measure the extracellular metabolites (appropriate standards added; lyophilised and store at −80°C prior to analysis). The sampling process to fixed samples took around 10 s. To monitor metabolite leakage, these samples were compared with lyophilised samples rapidly filtered through 0.2 µm pore size syringe filters 30 s later. After 30 s a further 1 mL of culture was sampled, degassed and added to 1 mL of ethanol in 2 mL pre-weighed centrifuge tubes. The samples were flash centrifuged (20,000 g; room temperature) and the supernatant removed. The cell pellet and extracellular metabolite samples were then lyophilised and cell pellet samples were weighed for the determination of biomass, although not determined here the biomass can be further digested to measure the biomass composition. A further 30 µL of culture was fixed with 70 µL ethanol (room temperature) for the determination of cell number and size.

**Figure 2:**
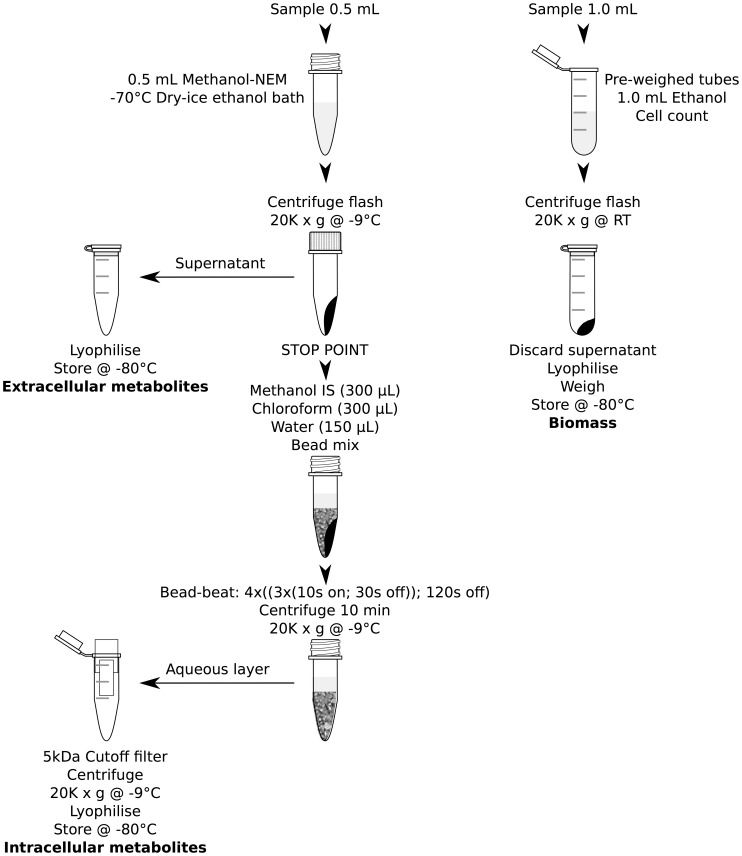
Extraction protocol flow diagram.

**Table 1:** Metabolites (attomol/cell) extracted from samples taken during respiratory oscillation (minimum dissolved oxygen; figure 4), detected by CE-MS and the methods used to extract them. nd – not detected.

**Name**	**Sonication**	**Sonication -Q** 1	**Freeze thaw**	**BB** 2, 3	**BB** 2 **-NEM** 4 **before** 5	**BB** 2 **-NEM** 4 **after** 6	**BB** 2 **-NEM** 4 **-Q** 1
*Pyruvate*	2.170±0.876	2.219±0.132	1.443±0.612	1.273	1.482±0.175	1.666±0.039	0.604±0.029
*Lactate*	1.653±1.187	2.495±0.069	0.381±0.124	1.339	0.949±0.064	1.261±0.107	1.437±0.088
*Fumarate*	1.607±0.617	2.180±0.144	1.096±0.657	0.683	0.629±0.040	0.626±0.061	0.473±0.002
*2-Oxoisopentanoate*	nd	nd	nd	0.145	0.178±0.009	0.154±0.020	0.091±0.006
*Succinate*	1.451±0.306	5.073±0.356	1.680±0.423	1.373	1.137±0.036	1.709±0.262	8.124±0.172
*Malate*	2.231±1.592	6.874±0.560	2.150±1.509	2.585	2.292±0.074	2.221±0.151	2.036±0.036
*2-Oxoglutarate*	nd	nd	nd	0.948	0.974±0.160	nd	nd
*PEP*	nd	nd	nd	0.220	0.429±0.079	0.363±0.002	0.193±0.017
*DHAP*	nd	0.984±0.210	nd	0.230	0.520±0.201	0.526±0.134	0.377±0.083
*Glycerophosphate*	1.269±0.275	2.175±0.321	1.390±0.989	0.629	0.613±0.021	0.672±0.125	0.719±0.062
*cis-Aconitate*	0.105±0.056	nd	0.141±0.068	0.521	0.365±0.007	nd	nd
*3PG*	3.825±2.763	nd	0.873±1.010	4.432	4.317±0.343	4.969±0.225	0.703±0.045
*Ru5P*	0.264±0.113	nd	nd	0.254	0.473±0.120	0.453±0.040	0.172±0.038
*G1P*	0.757±0.353	0.699±0.030	0.011±0.004	0.504	0.539±0.060	0.656±0.119	0.631±0.020
*F6P*	0.498±0.232	0.716±0.028	nd	nd	0.451±0.048	nd	nd
*G6P*	0.641±0.873	2.399±0.110	0.010±0.003	5.110	7.325±0.247	5.432±0.333	1.661±0.046
*2,3-DPG*	nd	nd	nd	0.032	0.045±0.011	nd	0.012±0.017
*6-Phosphogluconate*	0.441±0.246	0.299±0.047	nd	0.889	1.165±0.350	0.933±0.066	0.122±0.019
*S7P*	0.817±0.431	0.386±0.011	nd	0.843	0.951±0.035	0.677±0.070	0.103±0.002
*F1,6P*	1.160±0.810	7.337±1.831	0.011±0.004	0.547	0.908±0.033	0.730±0.172	0.797±0.064
*Citrate*	2.470±0.457	3.825±0.270	1.035±1.119	2.174	2.316±0.153	2.040±0.250	1.650±0.037
*CMP*	0.217±0.040	0.223±0.024	0.106±0.066	0.178	0.123±0.007	0.211±0.042	0.620±0.010
*AMP*	3.668±0.599	2.264±0.222	1.760±1.211	2.239	1.278±0.099	2.505±0.562	6.824±0.225
*IMP*	0.540±0.086	0.483±0.035	0.268±0.168	nd	0.083±0.017	nd	nd
*GMP*	0.475±0.081	0.292±0.037	0.171±0.151	0.233	0.155±0.014	0.279±0.071	1.576±0.004
*NADPH*	0.003	0.280±0.010	nd	nd	nd	nd	0.198±0.002
*CoA*	0.094±0.027	0.125±0.008	0.018±0.023	0.020	0.160±0.017	0.139±0.036	0.228±0.014
*dTDP*	0.014±0.002	0.016±0.002	nd	0.046	0.035±0.004	0.041	0.039±0.001
*CDP*	0.135±0.017	0.151±0.027	0.034±0.044	0.297	0.235±0.019	0.315±0.009	0.221±0.004
*Acetyl CoA*	0.025±0.002	nd	0.010±0.012	0.068	0.095±0.005	0.085±0.014	0.084±0.004
*ADP*	1.918±0.274	1.979±0.326	0.506±0.699	3.597	3.110±0.080	3.716±0.333	3.309±0.025
*GDP*	0.264±0.004	0.273±0.052	nd	0.358	0.320±0.020	0.400±0.035	0.712±0.012
*dCTP*	nd	nd	nd	0.034	0.039±0.006	0.035±0.004	nd
*dTTP*	0.006±0.001	0.012±0.002	nd	0.062	0.076±0.004	0.063	0.026
*CTP*	0.054±0.008	0.127±0.046	nd	0.601	0.739±0.009	0.569±0.030	0.151±0.005
*UTP*	0.262±0.027	0.216±0.047	0.074±0.102	1.635	1.939±0.065	1.509±0.027	0.550±0.008
*dATP*	0.005±0.001	0.007±0.001	nd	0.051	0.058±0.012	0.046±0.010	0.026±0.006
*ATP*	1.167±0.218	2.271±0.490	0.361±0.506	8.814	10.520±0.588	7.605±0.088	3.082±0.037
*GTP*	0.165±0.016	0.240±0.071	nd	1.774	2.027±0.112	1.654±0.123	1.037±0.008
*NAD+*	2.120±0.172	1.707±0.086	nd	3.504	3.711±0.107	3.639±0.013	3.358±0.082
*NADH*	0.025±0.003	2.421±0.313	nd	nd	nd	nd	0.964±0.149
*NADP+*	0.306±0.023	0.183±0.013	0.068±0.093	0.520	0.520±0.013	0.504±0.032	0.382±0.011
*FAD*	0.052±0.007	0.072±0.007	nd	nd	0.184±0.012	0.144±0.087	0.087±0.027
*Gly*	1.938±0.171	2.006±0.120	1.895±0.056	7.184	7.270±0.745	8.590±0.027	16.670±5.088
*Ala*	2.018±0.080	2.012±0.031	2.060±0.054	190.603	198.371±16.626	228.475±3.048	302.253±0.606
*GABA*	2.000±0.085	1.975±0.198	1.974±0.106	1.021	1.097±0.113	1.259±0.068	1.377±0.037
*2AB*	2.016±0.155	2.020±0.132	2.047±0.186	0.893	0.976±0.071	1.113±0.052	1.310±0.075
*Ser*	2.113±0.178	2.163±0.058	2.164±0.079	6.481	6.712±0.547	7.691±0.059	10.683±5.029
*Pro*	2.043±0.228	2.053±0.240	1.985±0.320	5.271	5.512±0.461	6.283±0.038	9.575±0.759
*Val*	1.871±0.315	2.087±0.123	2.059±0.097	38.476	43.287±3.415	48.741±1.008	55.543±1.200
*Homoserine*	2.362±1.049	1.714±0.121	1.742±0.134	1.691	1.971±0.168	2.127±0.082	1.410±0.042
*Thr*	2.500±1.057	2.567±0.899	2.854±1.111	12.228	13.525±1.159	14.918±0.192	16.956±0.692
*Cys*	nd	nd	nd	nd	0.310±0.049	0.343±0.022	0.444±0.008
*Ile*	2.042±0.119	2.045±0.162	2.072±0.032	4.053	4.592±0.456	5.340±0.084	6.518±0.618
*Leu*	2.103±0.143	2.139±0.197	2.112±0.228	1.567	1.589±0.114	1.913±0.045	3.025±0.766
*Asn*	2.009±0.124	2.034±0.095	2.050±0.134	3.447	3.920±0.304	4.161±0.124	5.038±0.168
*Ornithine*	1.924±0.087	1.933±0.071	1.865±0.061	9.902	8.199±0.726	8.850±0.796	10.901±2.848
*Asp*	2.004±0.090	1.955±0.121	1.969±0.050	13.221	55.773±1.133	56.621±0.508	55.545±2.242
*Homocysteine*	nd	nd	nd	0.132	0.709±0.097	0.737±0.006	0.795±0.036
*Adenine*	nd	nd	nd	0.151	0.172±0.007	0.155±0.007	0.139±0.003
*Gln*	2.008±0.113	2.046±0.143	2.013±0.155	104.444	223.521±10.435	228.527±0.257	225.379±0.076
*Lys*	2.003±0.136	2.009±0.095	2.036±0.144	12.354	34.010±1.118	35.580±0.021	35.572±0.776
*Glu*	1.980±0.129	1.977±0.115	1.997±0.121	835.237	864.465±19.172	887.669±10.922	886.352±2.001
*Met*	nd	nd	nd	0.454	0.487±0.061	0.554±0.012	1.003±0.033
*Guanine*	nd	nd	nd	0.068	0.071±0.001	0.066±0.006	nd
*His*	1.948±0.094	1.980±0.060	1.944±0.069	12.022	13.150±1.267	14.154±0.263	14.336±1.289
*Phe*	2.038±0.099	2.006±0.090	2.012±0.045	1.113	1.254±0.073	1.330±0.014	1.710±0.387
*Arg*	2.020±0.107	1.964±0.065	2.002±0.080	93.775	218.231±10.111	225.316±4.592	214.317±2.327
*Citrulline*	2.034±0.084	2.007±0.082	2.022±0.083	7.458	8.877±0.960	9.170±0.372	8.951±0.139
*Tyr*	2.114±1.093	2.076±0.968	2.374±1.191	1.227	1.400±0.090	1.509±0.017	1.843±0.584
*SAH*	1.932±0.257	1.910±0.223	1.987±0.283	0.318	0.330±0.020	0.407±0.002	0.696±0.007
*SAM+*	nd	nd	nd	0.530	0.549±0.028	0.553±0.079	0.532±0.008
*Trp*	2.060±0.099	2.053±0.069	2.058±0.124	0.254	0.287±0.015	0.287±0.028	0.365±0.073
*Cystathionine*	1.981±0.119	1.957±0.119	1.976±0.135	5.837	7.127±0.603	7.281±0.039	6.559±0.062
*γ-Glu-2AB*	nd	nd	nd	0.255	0.282±0.026	0.313±0.001	0.279±0.004
*γ-Glu-Cys*	nd	nd	nd	1.436	7.796±0.500	8.822±0.415	8.220±0.438
*Adenosine*	2.009±0.152	1.970±0.124	1.949±0.190	0.281	0.444±0.064	0.421±0.156	0.408±0.016
*Guanosine*	nd	nd	nd	0.108	0.125±0.005	0.131±0.022	0.090±0.008
*Glutathione(ox)*	2.103±0.279	2.078±0.243	2.162±0.308	13.105	14.840±0.114	15.060±0.199	14.559±0.086
*Glutathione(red)*	2.004±0.148	2.015±0.150	1.923±0.125	36.580	143.608±12.226	147.043±5.378	129.318±1.290

1 -Q not quenched.

2 BB bead-beating.

3 All other samples were performed in triplicate, except this one.

4 NEM *N*-ethylmaleimide.

5 NEM added pre bead-beating.

6 NEM added post bead-beating.

#### Determination of dry cell weight and cell number

Dry cell weight was calculated by weighing the tube and lyophilised pellet from the tube weight using an accurate balance (+/−0.1 mg, HR-202i, AND, Japan). Cell number and volume distributions were measured using a particle counter (50 µm orifice, CDA-500, Sysmex, Japan) by vortexing the ethanol fixed samples for 30 s and pipetting 2.5 µL into 19.9975 mL of Manoresh™ (Sysmex, Japan). The cell number and volume was used to calculate the average metabolite concentration (attomol/cell) for each sample.

**Figure 3:**
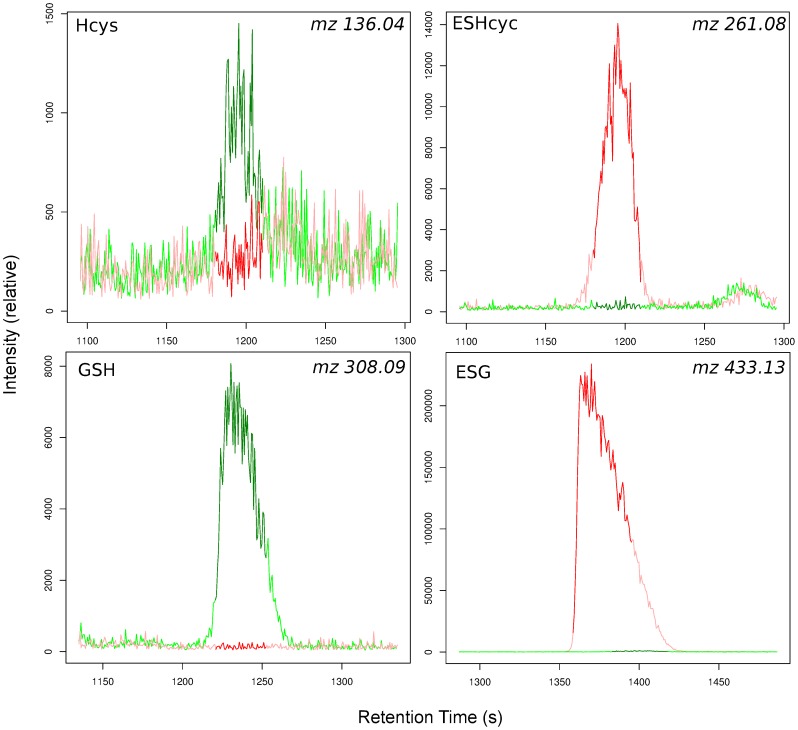
Electrophoretograms of glutathione (GSH) and homocysteine (Hcys) and their NEM derivatives (ESG and ESHcys) in representative samples with (red line) and without (green line) the addition of 2 mM NEM.

#### N-Ethylmaleimide bead-beating method

The cell pellets were resuspended in internal standards-methanol solution (300 µL; −70°C). The internal standards comprised of 2-morpholinoethanesulfonic acid (MES; 10 nmol), methionine sulfone (10 nmol) and D-camphor-10-sulfonic acid (CSA; 10 nmol). As the analysis volume was 50 µL, the final amount added of each standard was adjusted to a 200 µM solution on rehydration prior to analysis. Chloroform and deionised water (final ratio 1∶1∶0.5) was then added. A mixture of chilled acid washed zirconia/silica beads (∼300 µL; 0.1 mm and 0.5 mm; 1∶1; Tomy Seiko Co., Ltd., Japan) were then added and the samples were disrupted in a multi-tube bead-beater (5500 rpm; Micro Smash™ MS-100, Tomy, Japan), using 12 cycles of 10 s beat, 30 s rest (for cooling) and a further 120 s rest after every third cycle, with inbuilt refrigeration unit turned on. The samples were then centrifuged (12,000 g; 10 min; −9°C), and the aqueous phase (if a clear phase separation was not obtained more water can be added and the samples mixed then re-centrifuged) transferred to 5 kDa cut-off filter tubes (Ultrafree®-MC, Millipore, USA) and centrifuged (20,000 g; −9°C until the supernatant had passed through the filter). The filtration step removes many proteins and the filtrate was then lyophilised and stored at −80°C prior to CE-MS analyses. For comparison we also have extracted metabolites using different cell disruption methods and conditions: (1) sonication, 20 min, with and without quenching (2) freeze-thaw using liquid nitrogen, thaw at −30°C, 10 times (3) bead-beating without NEM treatment (4) NEM treatment after bead-beating, with and without quenching. Prior to analysis intracellular metabolite samples were rehydrated in 50 µL and extracellular samples were rehydrated in 500 µL of Milli-Q water, containing 200 µM 1,3,5-benzenetricarboxylate (anion) and 3-aminopyrrolidine dihydrochloride (cation).

### Capillary Electrophoresis Analytical Methods

In all CE-TOFMS experiments we used the Agilent CE capillary electrophoresis system (Agilent Technologies, Waldbronn, Germany), the Agilent G3250AA LC/MSD TOF system (Agilent Technologies, Palo Alto, CA), the Agilent 1100 series binary HPLC pump, the G1603A Agilent CE-MS adapter, and the G1607A Agilent CE-ESI-MS sprayer kit. Data acquisition was with G2201AA Agilent ChemStation software for CE and Analyst QS software for Agilent TOFMS (Agilent technologies, Japan). For anionic metabolite profiling, the original Agilent stainless steel ESI needle was replaced with the Agilent G7100–60041 platinum needle [23].

**Figure 4:**
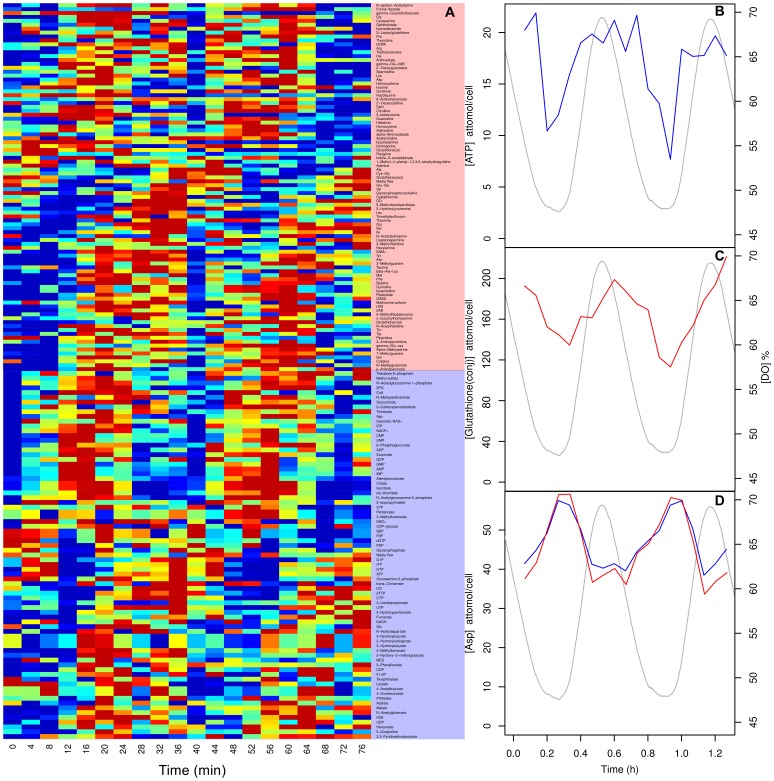
A heatmap of the calibrated intracellular metabolite time-series during the respiratory oscillation (A) and time-series for ATP (B), glutathione (C), and aspartate (D) in *S. cerevisiae*. Cationic and anionic data are shown in red and blue (lines or shaded areas), respectively. The corresponding oscillation in dissolved oxygen (DO) is represented by a grey line.

#### CE-TOFMS conditions for cationic metabolite profiling

Metabolites were separated in a fused silica capillary (50 µm i.d. x 100 cm) filled with 1 M formic acid as the electrolyte [4]. Prior to first use, a new capillary was flushed with the running electrolyte (1 M formic acid) for 20 min. A sample solution was injected at 5 kPa for 3 s (3 nL), and 30 kV voltage was applied. The capillary temperature and the sample tray were set at 20°C and below 5°C, respectively. Methanol:water (50% v/v) containing 0.1 µM hexakis (2,2-difluoroethoxy) phosphazene was delivered as the sheath liquid at 10 µL/min. ESI-TOFMS was in the positive ion mode, and the capillary voltage was set at 4 kV. The flow rate of heated dry nitrogen gas (heater temperature 300°C) was maintained at 170 kPa. At TOFMS, the fragmentor, skimmer and Oct RFV voltages were set at 75, 50, and 125 V, respectively. Automatic recalibration of each acquired spectrum was with reference masses of reference standards ([^13^C isotopic ion of protonated methanol dimer (2MeOH+H)]^+^; m/z 66.0632) and ([hexakis (2,2-difluoroethoxy) phosphazene+H]^+^; m/z 622.0290). Exact mass data were acquired at a rate of 1.5 spectra/s over a 50–1,000 m/z range [24].

#### CE-TOFMS conditions for anionic metabolite profiling

A cationic polymer-coated COSMO(+) capillary (50 µm i.d. x 110 cm) (Nacalai Tesque, Kyoto, Japan) was used as the separation capillary. A 50 mM ammonium acetate solution (pH 8.5) was used as the electrolyte for the capillary separation. Prior to first use, a new capillary was flushed successively with the running electrolyte, 50 mM acetic acid (pH 3.4), and then the electrolyte again for 20 min each. Before each injection, the capillary was equilibrated for 2 min by flushing with 50 mM acetic acid (pH 3.4), and then for 5 min with the running electrolyte. A sample solution (30 nL) was injected at 5000 kPa for 30 s, and −30 kV of voltage applied. 5 mM ammonium acetate in 50% (v/v) methanol-water containing 0.1 µM hexakis (2,2-difluoroethoxy) phosphazene was delivered as the sheath liquid at 10 µL/min. ESI-TOFMS was conducted in the negative ionization; the capillary voltage was set at 3,500 V. At TOFMS, the fragmenter, skimmer, and Oct RFV voltages were set at 100 V, 50 V, and 200 V, respectively. Automatic recalibration of each acquired spectrum was performed using reference masses of reference standards ([^13^C isotopic ion of deprotonated acetic acid dimer (2CH_3_COOH-H)]^-^, m/z 120.03841), and ([hexakis (2,2-difluoroethoxy) phosphazene + deprotonated acetic acid (CH_3_COOH-H)]^-^, m/z 680.03554). Other conditions were identical to those used in cationic metabolite analysis [23].

#### Data analysis

We first visualised the raw data in Analyst QS to check for any problems in the CE-MS. The data were then converted into mzXML format using mzWiff [25] for Agilent “.wiff” and trapper [26] for Agilent “.d” formats, using the following parameters:

mzWiff.exe -z -c1 -v –mzXML <filename>

trapper.exe -c -v –mzXML <filename>

Data was then loaded into the xcms package [27] of Bioconductor for further processing. CE-MS spectra tend to drift significantly as the column properties change with time [28]. This makes the inbuilt alignment algorithms in XCMS perform poorly. Therefore, peaks were pre-aligned using a regression analysis of the internal standards. The alignment was then carried out using XCMS for the datasets, where we used the matchedFilter method and set the following parameters, bw = 8, mzwid = 0.25, fwhm = 6, snthresh = 5 and step = 0.1. The concentrations were calculated using external standards that were prepared and lyophilised weekly, then reconstituted freshly prior to each run. This freshly prepared high quality set was a subset (61 anionic and 59 cationic) of the whole standard set (272 anionic and 318 cationic; prepared and lyophilised monthly) which was also run (data not shown).

## Results and Discussion

### Metabolome

The overall internal standard recovery rate (average for all the samples taken) for 2-(N-morpholino)ethanesulfonate (anionic) was 89.2%±2.32% and methionine sulfone (cationic) was 93.8%±3.8%. These standards were added prior to bead-beating therefore give an idea of the recovery from the extraction protocol. We used three methods to extract metabolites from yeast cells. Out of these, bead-beating performed the best (table 1) with a higher yield of most metabolites. Freeze thaw, although limiting the number of handling steps produced very low, variable yields. A striking example of this was the yield of ATP and amino acids, which were at least a magnitude higher using bead-beating. Quenching with methanol prior to extraction is known to cause metabolite leaching [8], however removing the cells from the media using centrifugation and to a lesser extent filtration causes a rapid changes in physiology [29]. Therefore, any quenching method is a trade-off. We examined the effect of quenching and found that the metabolites that increased between the filtered samples and the methanol-NEM supernatant were glutamate (5.03%), citrate (2.85%), succinate (8.34%) and pyruvate (12.34%) and these were within the error of the residual levels of the filtered samples. More significantly, flash centrifugation (10 s process) of the cultures caused very rapid metabolome-wide changes (table 1). For example [ATP] decreased from 10.5 to 3.1 attomol/cell, [AMP] showed a reverse trend increasing from 1.3 to 6.8 attomol/cell, suggesting ATP is rapidly utilised when the samples are centrifuged. There were also significant changes in TCA cycle intermediates where succinate increased, citrate decreased, and NADH became detectable when quenched and non-quenched samples were compared. This indicates that live cell pelleting may inhibit the respiratory chain, perhaps by limiting oxygen transfer.

### Thiol Analysis

Thiols are critical for the understanding of redox biochemistry and all the tested methods were not able to measure thiols in a consistent way, and produced much lower concentrations than expected. Therefore, we used NEM which binds to the -SH group of thiols, thus protecting it from oxidation (figure 1; cysteine, homocysteine, L-γ-glutamyl-L-cysteine and reduced glutathione) [19]. The spectra were compared to identify peaks that corresponded to NEM-conjugated thiols (+125.048 mass; figure 3). All of the thiol compounds were measurable with low error (table 1), in addition the peaks also tended to migrate slower. Cysteine and L-γ-glutamyl-L-cysteine could be detected and homocysteine and glutathione were present at ∼70 fold higher concentrations compared to either sonicated samples or non-NEM treated samples. NEM is not charged therefore excess NEM does not influence the CE-MS run. Samples were oxidised after extraction as thiol metabolite concentrations were similar for the addition of NEM pre or post bead-beating (table 1).

### Time-series Analysis

Next we tested our extraction methods during experimental conditions. When grown continuously, budding yeast synchronise their respiratory activity to form a robust respiratory oscillation [20], [30] whose period ranges from 35 min to 8 h [31]. We measured the intracellular metabolites for two cycles for *S. cerevisiae* (figure 4). We highlight three metabolites, ATP, glutathione and aspartate whose concentrations had previously been determined by other methods in *S. cerevisiae* under similar conditions [20], [32], [33]. Our extraction protocol produced reproducible time-series for all calibrated metabolites (figure 4A) many with a robust oscillation. ATP oscillated (figure 4B) with an amplitude of 7–20 attomol/cell (0.6–1.5 mM) which was in the range (1–2 mM) of recent online measurements using online nanosensors [34]. Glutathione-NEM conjugate oscillated (figure 4C) with an amplidude of 120–205 attomol/cell (8.4–14.2 mM) which was an order of magnitude higher than previously reported values for GSH (0.6–1.2 mM) extracted with perchloric acid during the oscillation [35]. We argue that this increase in yield was due to enhanced disruption, rapid quenching and thiol protection during the extraction process. Aspartate is an interesting example of a cross-platform replicate as it is an acidic amino acid therefore can be detected in CE-MS anionic and cationic detection modes (figure 4D). It oscillated in the range of 37–56 attomol/cell (2–4 mM), which was double previously reported values for hot water extraction of 1–2 mM [33], [36]. Perhaps this increase in yield was due to avoiding live cell centrifugation steps.

It is clear from the time-series data (figure 4) that sample-to-sample error is very low between extractions and CE-MS detection methods. Indeed, none of the data presented here was normalised to the CE-MS internal standards (these were only used to align chromatograms). When this normalisation is done, paradoxically, the data becomes slightly noisier, perhaps because of the errors introduced during small volume pipetting operations or integration.

### Conclusions

Here we present an extraction protocol for yeast metabolites that rapidly quenches metabolism using methanol-NEM at sub −40°C temperatures from a small volume of culture (0.5 mL). NEM was shown to protect biological thiols from oxidation in subsequent sample processing steps. The method was tested on samples taken during the respiratory oscillation and was found to be reproducible and give equivalent or higher yields than previous data, which used different extraction protocols and analytical techniques. No one analysis platform can cover the entire scope of the metabolome, but our method removes ion contamination from the media (making it compatible with CE-MS) and, although not tested, our method has the potential to be expanded to analyse biomass composition (acid hydrolysis of biomass followed by LC/GC-MS) and the non-polar metabolites (in the chloroform phase of the extraction by LC/GC-MS). We envisage our methods can be readily applied for the reliable extraction of metabolites from other microbial systems.
